# Awareness and Knowledge about Human Papilloma Virus Infection among Students at Secondary Occupational Health School in China

**DOI:** 10.3390/ijerph18126321

**Published:** 2021-06-11

**Authors:** Xin Wang, Taifeng Du, Xiaoling Shi, Kusheng Wu

**Affiliations:** Department of Preventive Medicine, Shantou University Medical College, Shantou 515041, China; xwang1995@126.com (X.W.); taifengdu@163.com (T.D.); 20xlshi@stu.edu.cn (X.S.)

**Keywords:** cervical cancer (CC), human papilloma virus (HPV) infection, secondary occupational health school (SOHS), cross-sectional study, awareness and knowledge

## Abstract

Cervical cancer (CC) is one of the most common causes of cancer-related deaths worldwide. CC is mainly caused by human papilloma virus (HPV), which can be prevented by vaccination. We conducted a cross-sectional study in secondary occupational health school (SOHS) through a questionnaire aimed to assess the awareness and knowledge regarding HPV infection of students. A total of 2248 students participated in the survey, 45.3% of them had heard about CC, while only 21.9% of them had heard about HPV; and 74.2% had no idea of the causal link between HPV infection and CC. Most participants displayed poor awareness and knowledge about HPV infection. The results suggested that age, grade, major, academic performance, etc. were correlated with higher awareness of CC, HPV and HPV infection (*p* < 0.05). In multivariable logistic analysis, third-grade students had the most increased awareness of CC (OR = 17.13, 95%CI: [8.11, 36.15]), HPV (OR = 6.59, 95%CI: [4.16, 10.43]) and HPV vaccine (OR = 2.78, 95%CI: [1.78, 4.32]) when compared to first-grade. Awareness and knowledge regarding HPV infection were insufficient among students in SOHS. As the future healthcare providers, these results highlight the need to supplement targeted education to improve their awareness and knowledge of HPV and vaccination.

## 1. Introduction

The cervix is the organ connecting the uterus and the vagina, in which cervical cancer (CC) develops. CC is the fourth most common cancer for women worldwide, of which annual absolute estimates of new cases and deaths were 569,800 and 311,400 in 2018 [1]. More than 85% of cases occur in developing countries and China ranks 2nd behind India among countries in the death count of CC [1]. CC has caused substantive human, social and economic loss and become one of the emphases on prevention and control of cancer in long-term strategy of China.

Persistent HPV infection is the most important and necessary cause of almost all cases of CC [2]. HPV infection is often asymptomatic and the most common sexually transmitted infection (STI) [3]. In addition, there are other patterns of transmission such as hands to genitals [4]. HPV can infect epithelial cells of skin and mucosa, and cause genital warts and other cancers of oropharynx, vagina, anus vulva and penis [5,6]. There are more than 200 subtypes of HPV [7] of which subtypes of HPV 16 and 18 belong to high risk group and are associated with more than 80% of CC cases [8] as well as other cancers. Conversely, subtypes 6 and 11 are the most common low-risk HPV and related with genital warts [9]. Fortunately, HPV vaccines play an extremely important role in protecting against HPV infection especially oncogenic ones before any sexual activity. Since the year of 2016, three kinds of HPV vaccines (bivalent Cervarix^®^, quadrivalent Gardasil^®^ and nonavalent Gardasil^®^) have been approved in succession for use in mainland China [10].

Nevertheless, there is no sufficient data about the coverage rate of HPV vaccination due to the lack of national population-based plan on HPV vaccination in mainland China. It is Hong Kong the first place to license an HPV vaccine around China, and yet the uptake rate (7.2%) of Cervarix^®^ there was very low among adolescent girls [11]. A review also showed that the coverage rates of HPV vaccine in China were relatively low (2.4–9.1%) compared to other countries [12]. Lacking of awareness and knowledge about HPV infection was one of the main reasons and determinants for the low acceptance and uptake rates of HPV vaccine [11,13]. According to the document of World Health Organization, sexually non-active young people aged 9–13 years should be the targets for HPV vaccination. In mainland China, females more than 16 years old mostly have sexual experience [14]. Students at SOHSs, the targets of HPV vaccine, will be healthcare providers and they play very effective roles in promoting HPV vaccination in the future. Due to the recent introduction of HPV vaccine in China, it is necessary to assess the awareness and knowledge about HPV infection among these students. However, studies on the awareness and knowledge regarding HPV infection among this group are still lacking in China.

Hence, the aim of this study was to assess the awareness and knowledge about HPV infection among students at SOHS and identify the influencing factors to help design pertinent intervention measures for this population.

## 2. Materials and Methods

This study was a cross-sectional survey conducted between September and December 2019, targeting Chinese SOHS students. The sample size of this study was calculated using a rate of 16.8% of students aware of HPV which was acquired from pilot studies, a marginal error of 3% and 95% confidence interval. Considering a low response rate and design effect, there was a 30% increase in the sample size which was eventually determined as 776. A two-stage sampling was performed to recruit the participants. First, stratified sampling was conducted for medical and non-medical classes. Then, cluster sampling was employed to the selected classes. Finally, the participants consisted of 2248 students comprising 428 boys and 1820 girls responded to the survey carried out in the classrooms based on the WeChat platform Application (App).

The questionnaire of HPV infection was developed by the research team with the advice of experts. It was consisted of two domains: socio-demographic characteristics of participants were assessed with sixteen items; ten questions assessed awareness and knowledge regarding HPV infection among respondents. For Q1–7, Q9, Q10, responses of “Not Sure” were recoded as “No” in the statistical analysis. For Q8, it was with multiple choices and every option was correct. In this study, awareness in the questionnaire meant that the participants had heard about cervical cancer, HPV and HPV vaccine. Knowledge meant more details about HPV infection in the rest of the second domain of the questionnaire. These data were used to explore factors associated with HPV awareness and knowledge.

This study was approved by Ethical Review Committee of Shantou University Medical College, Shantou, China (SUMC-2019-62). Informed consents were collected from participants in our study and all responses would be confidential.

Data were analyzed using SPSS 26.0 (IBM, Chicago, IL, USA) and STATA 14.0 (Stata Corporation, College Station, TX, USA). To evaluate the data, descriptive statistics, percentage, frequency, mean and standard deviation were used, as appropriate. The chi-square test was used to analyze the differences. Multivariate logistic regression analysis was undertaken to assess the relationship between awareness, knowledge and characteristics among the respondents, and odds ratios (ORs) along with 95% confidence interval (CI) were also calculated. Only significant variables remained in the models. For all the analyses above, *p* < 0.05 was considered as the significance level.

Declaration: We used ‘AIDS’ incorrectly in order to be able to communicate the commonly used term to the subjects of the study.

## 3. Results

### 3.1. Demographic Characteristics of Participants

In this study, data of 2248 students were collected and analyzed with a 100% response rate. Most of the participants were female (81.0%), younger than 16 years old (60.5%) and lived in rural (73.0%). The majority of students (86.8%) were majored in the medical specialty. More than half of students were in their first-year (52.8%), followed by second-year (42.9%) and third-year (4.3%). Most students had relatively lower personal, paternal and maternal educational status, family income and academic performance. About 4.9% of the study populations had sexual experience. Among them 30 respondents (1.3%) had the history of CC in the family and 287 participants (12.8%) had the family history of other cancers. For all of them, 754 respondents (33.5%) had received at least one dose of the HPV vaccine (Table 1).

### 3.2. Awareness and Knowledge Questions about HPV Infection

Generally, awareness and knowledge on HPV infection were insufficient. In this study, it was found that 1019 respondents (45.3%) had heard about CC; 492 respondents (21.9%) had heard about HPV and 555 respondents (24.7%) had heard about HPV vaccine. Mostly, 1768 respondents (78.6%) didn’t know that men could be infected by HPV. Similarly, more than seventy percent of the participants didn’t know that HPV infection could cause CC and some other cancers. Their ignorance regarding the link of genital warts and liquid-based cytology of HPV infection should be taken seriously. Only 192 students (8.5%) realized that it was asymptomatic after HPV infection. Almost all the respondents (96.8) knew that AIDS was a STI, while only about half (51.6%) of the participants considered that HPV infection as STI, followed by syphilis (45.5%), gonorrhea (43.4%), fungal diseases (26.3%), herpes (24.2%) and hepatitis B (19.5%) (Table 2).

### 3.3. Sources of Information regarding HPV Infection

**Figure 1:**
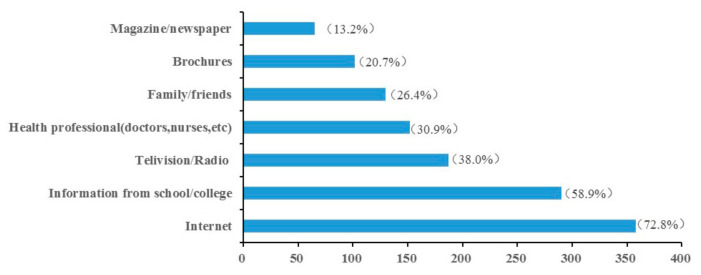
The sources of information the participants achieved for those who have heard HPV (Human Papillomavirus).

Among those (*n* = 492) who had heard about HPV, the majority (72.8%) had access to HPV-related information from the Internet, followed by their education (58.9%), television or radio (38.0%), health professional (30.9%), family or friends (26.4%), brochures (20.7%) and magazine or newspaper (13.2%) (Figure 1).

### 3.4. Awareness and Knowledge Regarding HPV Infection Stratified for Characteristics

In univariate analyses, female students were more likely to have heard about CC, knew that HPV could cause CC and other cancers compared to male students (*p* < 0.001). As for the respondents who were in higher ages, grades, majored in medical specialty and (or) had better academic performances, they knew more about Q1–7, Q9 and Q10 (*p* < 0.05). However, there was no difference in the awareness and knowledge of Q1–7, Q9 and Q10 between rural and urban residents. Among students themselves with higher education level, awareness and knowledge concerning Q1–7 and Q10 were more than their counterparts, while there was no difference in knowledge about that HPV infection was often asymptomatic. Paternal educational status reached significance with Q2, Q7, Q9 and Q10, whereby students whose father had higher education level were more likely to be aware of HPV and knew that HPV could cause genital warts, HPV infection was often asymptomatic and could be detected by liquid-based cytology. Maternal higher educational status was associated with the students’ increased awareness and knowledge of HPV, HPV vaccine, availability for HPV of men and methods for preventing HPV infection compared to their counterparts. The students with higher family income more endorsed knowledge of HPV vaccine and HPV’s relation to men and CC. Significant differences in characteristics of sexual experience and family history of CC were found in the responses to Q1, Q2 and Q9. It was showed that there was statistically significant association between the characteristic of family history of other cancers and the responses to Q1–7. HPV-vaccinated participants demonstrated significantly better awareness and knowledge of Q2–7, Q9 and Q10 (Table 3).

### 3.5. Multivariate Logistic Regression Analysis

**Figure 2:**
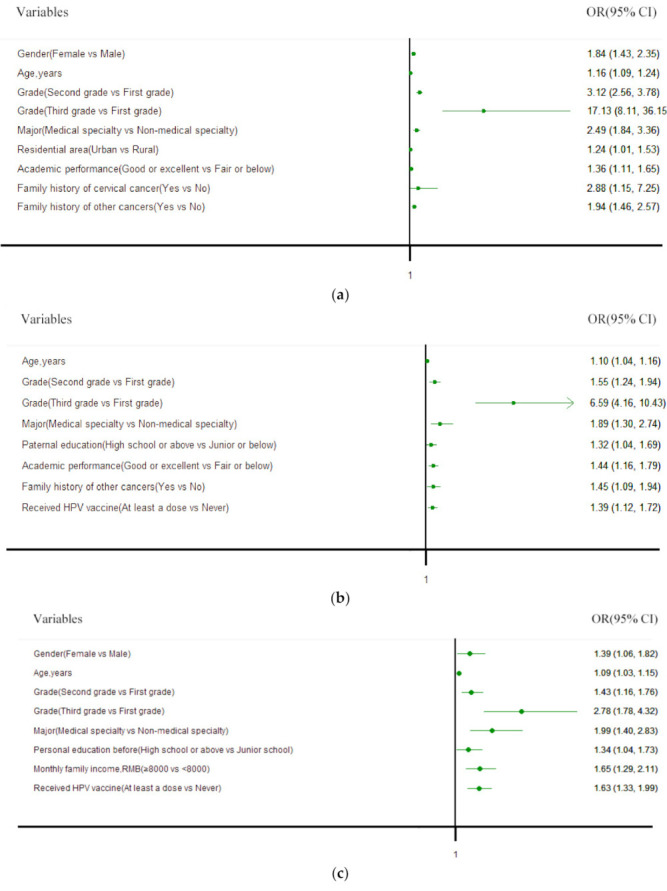
The forest plots of multivariable logistic regression analysis for awareness of: (**a**) CC (cervical cancer); (**b**) human papillomavirus (HPV); (**c**) the HPV vaccine.

The multivariate logistic regression analysis indicated that female students were more likely to be aware of CC (OR = 1.84, 95%CI [1.43, 2.35]) and HPV vaccine (OR = 1.39, 95%CI [1.06, 1.82]) than males. Increased awareness of CC, HPV and HPV vaccine was significantly associated with older students (respectively, OR = 1.16, 1.10, 1.09, for every 1-year increment, *p* < 0.05). The strongest association between characteristics and the awareness of CC, HPV and HPV vaccine was grade. The three models revealed that those students with higher grades and (or) majored in medical specialty were more likely to be aware of CC, HPV and HPV vaccine (*p* < 0.05). Urban dwellers were 1.34 times more likely to be aware of CC than rural residents (OR = 1.34, 95%CI [1.01, 1.53]). The participants with higher education before enrollment were more likely to know HPV vaccine (OR = 1.34, 95%CI [1.04, 1.73]). The respondents whose father with higher education were more likely to realize HPV. Students with higher family income were 1.65 times more likely to have heard of HPV vaccine. Compared to those whose academic performances were fair or below, participants with better academic performances were more likely to be aware of CC (OR = 1.36, 95%CI [1.11, 1.65]) and HPV (OR = 1.44, 95%CI [1.16, 1.79]). The first model also revealed that subjects having family history of CC (OR = 2.88, 95%CI [1.15, 7.25]) and other cancers (OR = 1.94, 95%CI [1.46, 2.57]) were associated with higher odds of awareness of CC than counterparts. Students with family history of other cancers were 1.45 times more likely to know HPV compared to their counterparts. HPV-vaccinated participants were more likely to be aware of HPV (OR = 1.39, 95%CI [1.12, 1.72]) and HPV vaccine (OR = 1.63, 95%CI [1.33, 1.99]) (Figure 2).

## 4. Discussion

To the best of our knowledge, this is the first study implemented in southern China to measure the awareness and knowledge of students in SOHS regarding HPV infection. It is hard to compare our results with previous studies due to the lack of surveys in the same group. We acquired a good response of 100%, which suggested that the results could represent this population. Most of students were female, majored in medical specialty, lived in rural area and had no sexual experience. We found the vaccination rate of HPV vaccine in our participants was lower than that found in another study of Germany conducted in 2011, where 67% of female students aged 18–20 years were vaccinated [14] and lower than a national survey of America in 2016 which reported 43.4% of adolescents aged 13–17 years had been vaccinated [15]. Of note, this indicated the necessity of better education to implement HPV vaccine in mainland China.

The recruited students had relatively low level of awareness and knowledge regarding HPV infection (21.9%), which was far below than studies conducted in Spain (54.34%) [16] and Italy (all) [17]. The present study revealed that almost half, 1019 (45.3%) of the respondents had heard about CC, higher than a prior study (35.4%) in Jinan, China [18]. Notably, only about a fifth to a quarter of the participants were aware of HPV and HPV vaccine. The awareness rates of HPV and HPV vaccine were in line with a study [18] and a meta-analysis of China [19], while the rates were lower than their overseas peers [14,20]. In addition, the rate of awareness of HPV was similar to another two studies in China [21,22]. The results suggested that the students had a high awareness level regarding CC, but awareness rate of HPV was relatively low. Similar results were observed in a published literature [23,24]. In this study, the rates of the participants who knew that HPV could cause CC and genital warts were 25.8% and 22.4%. A study conducted in Turkey among people more than 17 years old, reported that corresponding rates were 13% and 16.7% [25]. The rates of the awareness of CC, HPV and HPV vaccine were higher than peer students in China [18], which might be due to the fact that most of our respondents were majored in medical specialty. An American study demonstrated the two rates above were 39% and 33.8% [26] and another study conducted in Italy showed 46.6% of the respondents knew the causal link between HPV infection and CC [27]. Generally, it seemed that our results were more positive than Turkey but less positive than some developed countries. This might be due to the fact that the implementation of HPV vaccine in developed countries was earlier than China and the general characteristics of the study groups were different. These results suggested that we should not only increase the awareness of HPV but also emphasize the causality among CC, HPV and HPV vaccine. Of the seven STIs questioned, the results indicated the students knew the best about AIDS, while only half of them knew that HPV infection was a kind of STIs. This reflected that the inadequate education about HPV infection in comparison to AIDS, even though the prevalence of HPV was higher than that of AIDS among the adolescents [28]. Of the respondents, the great majority didn’t realize that HPV infection was most often asymptomatic (8.5%) and could be detected through liquid-based cytology (15.6%). The rates of correct answers of these two questions were the lowest, similar to a previous literature [14]. Many of the respondents did not know that HPV infection could cause CC, genital warts, was often asymptomatic and could be detected by liquid-based cytology. 

Further analysis revealed that the Internet was their first source of information about HPV infection among participants aware of HPV, while previous studies reported the reverse results [29,30,31]. It suggested that the Internet played an increasingly important part in HPV-related information dissemination. As students mostly majored in medical specialty, education system was also an essential source of HPV related information, as published researches [32,33]. However, information from the Internet was uneven and HPV-related knowledge was insufficient among the healthcare providers [34], which mostly helped to translate the knowledge into concrete action. Most of our participants would be the future healthcare providers and therefore there was much potential for improvements in the medical education curriculum. For general population, our results inspired that we could invite experts or social media influencers to make some interactive and informative videos and promote them on the Internet.

In our study, female students knew more about CC and HPV-related cancers compared to males, while there was no sex difference in awareness of HPV and HPV vaccine and knowledge of other detailed information about HPV, which was similar to a study among the junior middle school students [18]. However, a published literature showed that women knew more than men in knowledge about HPV infection and HPV vaccine [25]. Due to the tendentiousness of these cancers, females might have more opportunities to get related information from outside. The HPV vaccine had just been introduced in China not very long and participants in the survey might squeeze information from the Internet and education system at the same chance. The respondents younger than 16 years had a relative low awareness and knowledge in those nine questions, which might attribute to more potential to pick up information. Our results also revealed that students with higher grades had a better understanding of the questions, which was in line with an India survey [33], and this might be owed to the provision of HPV-related courses [35]. Students majored in medical specialty and (or) had a better academic performance did well in all questions above, similar to previous literatures [35,36]. Different from a previous study [37], there was no difference in any question above among students in different residential areas, which might be because of the popularization of the Internet and the narrowed gap of our respondents in awareness and knowledge of HPV infection. We observed that parents’ education level had difference in some of questions above, which indicated that the improvement of the parents’ education level might take effect in increasing the knowledge among them [38]. The results revealed that sexual experience didn’t show much difference and thus it was essential to improve their knowledge level and coverage rate of HPV vaccine before any sexual activity. Our observation that monthly income also didn’t make much difference as previous study reported [21]. The respondents with family history of CC were more potential to heard about CC and HPV. Additionally, the characteristics of personal education level, family history of other cancers and having received HPV vaccine suggested much difference in questions above, similar to previous surveys [21,37]. The results of the three multivariable logistic regression models showed that age, grade and major were three independently significant predictors. Indeed, students with higher ages, grades and medical specialty were more likely to have heard about CC, HPV and HPV vaccine. As a rule of thumb, participants with higher age and education and medical specialty had more opportunity to obtain HPV-related information, consistent with published studies [5,39]. Thus, results in this study suggested pertinent education programs and then conceptualized HPV as an infectious agent with different potential severity and consequences among the participants. 

The limitations of this study are as followed. Firstly, the study found a number of correlations between the level of HPV knowledge with the characteristics of respondents, but explaining causality needs to be further explored based on research features. Secondly, as the participants filled in the questionnaire themselves, there was partial recall bias. Thirdly, the participation of the third-grade students was obviously lower than the other two grades, which was because the majority of them were on an internship outside school. Finally, this survey was only aimed at students in health schools. Multiple schools and groups are planned to be included in the survey to elucidate the cognitive level and possible influencing factors in the later stage.

## 5. Conclusions

In conclusion, the results underscore that the overall awareness and knowledge regarding HPV infection among students in SOHS is insufficient and superficial. As most of the respondents will be the future healthcare providers, it is crucial to equip them with professional and adequate knowledge through targeted education campaigns and curriculums for the implementation of HPV vaccine and prevention of CC among them and more general population.

## Figures and Tables

**Table 1:** Socio-demographic characteristics of the study respondents (*n* = 2248).

Variables	Responses	N (%)
Gender	Male	428 (19.0)
	Female	1820 (81.0)
Age (years)	16 [16, 17] (13–42) ^1^	2248 (100)
	<16	1360 (60.5)
	≥16	888 (39.5)
Grade	First grade	1187 (52.8)
	Second grade	965 (42.9)
	Third grade	96 (4.3)
Major	Non-medical specialty	296 (13.2)
	Medical specialty	1952 (86.8)
Residential area	Rural	1640 (73.0)
	Urban	608 (27.0)
Personal education before	Junior school	1845 (82.1)
	High school or above	403 (17.9)
Paternal education	Junior school or below	1766 (78.6)
	High school or above	482 (21.4)
Maternal education	Junior school or below	1900 (84.5)
	High school or above	348 (15.5)
Family income, monthly (RMB)	<8000	1851 (82.3)
	≥8000	397 (17.7)
Academic performance	Fair or below	1470 (65.4)
	Good or excellent	778 (34.6)
Sexual experience	No	2138 (95.1)
	Yes	110 (4.9)
Family history of cervical cancer	No	2218 (98.7)
	Yes	30 (1.3)
Family history of other cancers	No	1961 (87.2)
	Yes	287 (12.8)
Received HPV vaccine	Never	1494 (66.5)
	At least a dose	754 (33.5)

^1^ Median [quartile 1, quartile 3] (range). 1 RMB = 0.14 US Dollar.

**Table 2:** Awareness and knowledge about HPV infection among the study participants (*n* = 2248).

Variables	Response	N (%)
Q1: Heard about cervical cancer	Yes	1019 (45.3)
Q2: Heard about HPV	Yes	492 (21.9)
Q3: Heard about the HPV vaccine	Yes	555 (24.7)
Q4: Men can get HPV	Yes	480 (21.4)
Q5: HPV can cause cervical cancer	Yes	580 (25.8)
Q6: HPV can cause other cancers	Yes	528 (23.5)
Q7: HPV can cause genital warts	Yes	503 (22.4)
Q8: Which of these are STIs	AIDS	2177 (96.8)
	Syphilis	1022 (45.5)
	Gonorrhea	976 (43.4)
	Fungal diseases	592 (26.3)
	HPV infection	1161 (51.6)
	Herpes	545 (24.2)
	Hepatitis B	439 (19.5)
Q9: HPV infection has no visible signs or symptoms	Yes	192 (8.5)
Q10: HPV can be detected by liquid-based cytology	Yes	350 (15.6)

STIs, Sexually Transmitted Infections.

**Table 3:** The participants’ positive answers to some items of HPV infection stratified by characteristics (chi-square test).

Variables	Response	N	Q1	Q2	Q3	Q4	Q5	Q6	Q7	Q9	Q10
Gender	Male	428	146 ***	87	90	77	80 ***	70 ***	82	46	61
	Female	1820	873	405	465	403	500	458	421	146	289
Age (years)	<16	1360	515 ***	252 ***	296 ***	259 **	312 ***	296 *	254 ***	97 **	176 ***
	≥16	888	504	240	259	221	268	232	249	95	174
Grade	First grade	1187	347 ***	184 ***	229 ***	204 ***	247 ***	238 ***	219 ***	80 **	142 ***
	Second grade	965	584	248	280	247	285	256	244	99	170
	Third grade	96	88	60	46	29	48	34	40	13	38
Major	Non-medical specialty	296	73 ***	36 ***	41 ***	33 ***	41 ***	39 ***	31 ***	13 **	21 ***
	Medical specialty	1952	946	456	514	447	539	489	472	179	329
Residential area	Rural	1640	727	348	399	339	419	374	362	143	254
	Urban	608	292	144	156	141	161	154	141	49	96
Personal education before	Junior school	1845	782 ***	367 ***	419 ***	363 ***	458 *	413 **	385 ***	150	274 *
	High school or above	403	237	125	136	117	122	115	118	42	76
Paternal education	Junior school or below	1766	794	363 ***	423	363	443	402	377 *	139 *	259 *
	High school or above	482	225	129	132	117	137	126	126	53	91
Maternal education	Junior school or below	1900	855	399 *	453 *	390 *	477	443	420	153	282 *
	High school or above	348	164	93	102	90	103	85	83	39	68
Family income, monthly (RMB)	<8000	1851	822	393	422 ***	380 *	462 *	432	403	151	284
	≥8000	397	197	99	133	100	118	96	100	41	66
Academic performance	Fair or below	1470	587 ***	268 ***	324 ***	268 ***	319 ***	308 ***	292 ***	106 **	208 *
	Good or excellent	778	432	224	231	212	261	220	211	86	142
Sexual experience	No	2138	955 **	457 *	527	450	545	499	469 *	176 *	330
	Yes	110	64	35	28	30	35	29	34	16	20
Family history of cervical cancer	No	2218	996 ***	480 *	544	474	570	517	493	186 *	343
	Yes	30	23	12	11	6	10	11	10	6	7
Family history of other cancers	No	1961	839 ***	407 **	468 *	398 **	479 ***	429 ***	406 ***	160	297
	Yes	287	180	85	87	82	101	99	97	32	53
Received HPV vaccine	Never	1494	660	295 **	320 ***	295 **	347 ***	311 ***	295 ***	101 ***	197 ***
	At least a dose	754	359	197	235	185	233	217	208	91	153

* *p* < 0.05; ** *p* < 0.01; *** *p* < 0.001.

## Data Availability

All data generated or analyzed during this study are included in this article.
